# Self‐reported sleep pattern and recurrence of atrial fibrillation after catheter ablation

**DOI:** 10.1002/clc.23975

**Published:** 2023-01-17

**Authors:** Jiehui Cang, Naiyang Shi, Didi Zhu, Yaowu Liu, Qianxing Zhou, Long Chen

**Affiliations:** ^1^ Department of Cardiology, Zhongda Hospital, School of Medicine Southeast University Nanjing China; ^2^ Department of Epidemiology and Health Statistics, School of Public Health Southeast University Nanjing China; ^3^ Key Laboratory of Environmental Medicine Engineering, Ministry of Education, School of Public Health Southeast University Nanjing China

**Keywords:** atrial fibrillation, catheter ablation, hypnotics, recurrence, sleep pattern

## Abstract

**Background:**

Increasing evidence has shown the relationship between sleep and the recurrence of atrial fibrillation (AF). However, the association of different sleep patterns with AF recurrence after catheter ablation was rarely studied. We aimed to assess the role of different sleep behaviors in the risk of AF recurrence after catheter ablation.

**Methods and Results:**

A total of 416 consecutive participants from Zhongda hospital of Southeast University were finally analyzed. Sleep patterns were defined by chronotype, sleep duration, insomnia, snoring, and daytime sleepiness. A total of 208 patients (50.0%) had a healthy sleep pattern within a mean follow‐up of 32.42 ± 18.18 months. The observed number of patients with AF recurrence was 10 (50.0%), 80 (42.6%), and 40 (19.2%) in unhealthy, intermediate and healthy sleep groups, respectively (*p* < .01). After adjusting covariates, unhealthy sleep pattern was significantly associated with AF recurrence [hazard ratio = 3.47 (95% confidence interval CI: 1.726–6.979, *p* < .001)]. Sleep disorders such as inadequate sleep time (time <7 h or >8 h), insomnia and excessive sleepiness during daytime were associated with a higher risk of recurrence. Otherwise, improvement in sleep seemed to be associated with decreased risk of AF recurrence.

**Conclusion:**

This retrospective study indicates that adherence to a healthy sleep pattern is associated with a lower risk of AF recurrence. Also, improved sleep before ablation is associated with a lower risk of AF recurrence.

## 1 INTRODUCTION

Atrial fibrillation (AF) is featured by absolutely irregular heart rhythm and can lead to a significantly increased risk of stroke and heart failure, which is associated with substantial morbidity, mortality, and economic cost.^1^, ^2^, ^3^ Catheter ablation (CA) can effectively achieve rhythm control by ablating triggers and modifying atrial substrates with different forms of energy.^4^ However, a significant decline in freedom from AF remains to be a challenge. A meta‐analysis showed that the 62‐month success rate of a single CA procedure was only 59%.^5^

Previously identified lifestyle factors such as alcohol intake,^6^ smoking,^7^ and obesity^8^ are associated with AF recurrence. Though sleep instability may be associated with the risk of recurrent AF, the specific association between sleep and the risk of AF recurrence is not unclear.^9^ Emerging evidence has associated several sleep behaviors, such as excessive daytime sleepiness,^10^ sleep quality,^11^ sleep duration,^12^ and insomnia with episodes of AF. Sleep disorders can alter the activation of sympathetic nerves and increase inflammation and oxidative stress, which would also increase the risk of AF recurrence.^9^ Notably, different sleep behaviors are intrinsically linked, so different trials which focus on the same sleep behavior would show contradictory results.^13^ Theoretically, It is more reasonable to pool different sleep behaviors together when exploring the influence of sleep on the recurrence of AF.

Li et al. previously put forward a new sleep pattern index score that integrates 5 different sleep behaviors: chronotype, sleep duration, excessive daytime sleepiness, snoring and insomnia.^14^ Different from the previous Pittsburgh Sleep Quality Index (PSQI), the sleep pattern score is more succinct and easier for patients' follow‐up. What's more, it contains an evaluation of chronotype and daytime sleepiness, which is not included in PSQI.

Sleep pattern was proved to be significantly associated with episodes of cardiovascular diseases and incident arrhythmias.^14^, ^15^ However, the association between sleep pattern and recurrent AF postprocedure is still unknown. In the current study, we aimed to retrospectively analyze the predictive role of newly developed sleep pattern index score in the recurrence of AF among patients who underwent AF ablation.

## 2 METHODS

### 2.1 Study population

This is a retrospective and single‐center study. Patients hospitalized in Zhongda Hospital of Southeast University and receiving successful CA (both radiofrequency and cyro‐balloon ablation) were all included in this study. Ablation was deemed successful in the absence of symptomatic or asymptomatic atrial tachyarrhythmias lasting >30 s identified by surface electrocardiogram (ECG) or Holter monitoring after the blanking period (3 months). We excluded patients: (i) New York Heart Association functional class IV; (ii) unstable angina or acute myocardial infarction within 3 months; (iii) chronic obstructive pulmonary disease; (iv) severe chronic renal or hepatic impairment; (v) thyroid dysfunction; (vi) rheumatic heart disease; (vii) noninitial procedure; (vii) self‐reporting obvious fluctuation in sleep quality postprocedure.

### 2.2 Follow‐up and assessment of outcomes

All patients took uninterrupted oral anti‐coagulation and antiarrhythmia drugs for at least 3 months or long‐term with guidance from clinicians. Before the ablation procedure, antiarrhythmic drugs were usually discontinued ≥5 half‐lives before ablation, except for amiodarone. All patients were followed up in outpatient clinics or forms of telephone interviews. Patients who had a complaint about recurrence were asked to provide the first ECG or Holter monitoring which recorded the rhythm of AF, atrial flutter or atrial tachyarrhythmias. And for those who had no complaints about symptoms associated with recurrence, we performed a clinical assessment of recurrence, ECG and Holter monitoring to check AF recurrence. Follow‐up time was defined from the data of the procedure to the date of recurrence. While for those without recurrence, follow‐up time was defined from the date of the procedure to the date of the latest follow‐up.

We also collected information on patients who tried to improve sleep quality with longer‐use of hypnotics. “Longer‐use” was defined as taking hypnotics at least 5 days a week. Improvement recorded by case history or self‐reported improvement was recorded.

### 2.3 Assessment of sleep behaviors

Sleep behaviors were collected through a questionnaire. Chronotype was assessed by the question, “Do you consider yourself to be: (i) definitely a ‘morning’ person; (ii) indefinitely a ‘morning’ person.” Sleep duration was reported as the hours of sleep every 24 h (including naps). Insomnia symptoms were obtained by the question, “Do you usually have trouble falling asleep at night or do you wake up in the middle of the night?” with choices provided: (i) Yes; (ii) No. Snoring was asked by the question “Does your partner or a close relative or friend complain about your snoring?” with responses: (i) yes; or (ii) no. Daytime sleepiness was asked by the question, “Do you often doze off or fall asleep during the daytime when you don't mean to? (eg, when working, reading or driving)” with the choices provided: (i) Yes; (ii) No.^14^, ^15^

The final score for the sleep pattern was calculated by pooling 5 different sleep behaviors. Each sleep factor was coded 1 if meeting the healthy criteria and 0 if not. A higher score indicates a healthier sleep pattern.

### 2.4 Assessment of other covariates

Demographic and lifestyle behaviors such as age, gender, preexisting conditions, drugs, smoking, alcohol intake, systolic blood pressure, diastolic blood pressure, glucose, and information associated with CA procedures were recorded according to electronic hospitalization systems. During follow‐up in an outpatient clinic, some lifestyle behaviors such as exercise, smoking, and alcohol intake were checked again by asking patients directly. Measurements such as the size of the left atrial, and left ventricular ejection fraction were recorded according to echocardiography results before the CA procedure. Besides, early recurrence was also recorded.

### 2.5 Statistical analyses

Descriptive statistics were used to summarize patient characteristics. Baseline characteristics of the study participants were summarized across the healthy sleep score as mean ± *SD* or median (interquartile range) for continuous variables and *n* (%) for categorical variables, if appropriate. Wilcoxon's test or independent‐sample *t*‐test was performed to compare continuous variables in different groups, as appropriate. *χ* ^2^ test or Fisher's exact test was performed to compare categorical variables. Statistical tests were based on a two‐sided significance level of 0.05.

The analyses of time‐to‐clinical recurrence events were described by Kaplan–Meier curves and comparisons between the groups were performed by log‐rank test. Cox proportional hazard models were used to estimate the hazard ratios (HRs) and 95% Poisson confidences (confidence interval [CIs]) for all initial predictors of the incidence of AF recurrence. Cox regression was imputed for univariable analyzes to assess potential predictors. Variables that were statistically significant in univariable analysis and those which were non‐statistically significant but had a clinical significance (including AF type, age, gender, hypertension, diabetes, coronary artery disease, heart failure, β‐blocker, and LAD) were all included in further multivariable analysis. A “Forward: likelihood” method was applied in multivariable analysis. When we performed a Cox analysis of different sleep behaviors, the same method was applied.

The IBM SPSS Statistics 25.0 software and R statistics were used to perform statistical analyses.

## 3 RESULTS

### 3.1 Study population

After excluding 234 patients, a total of 416 patients were included in this study (Figure S1). The baseline characteristics of included patients were presented in Table 1A. Considering the number of study participants, we finally divided patients into three groups according to their final score index: 0–1 (unhealthy sleep pattern), 2–3 (intermediate sleep pattern), 4–5 (healthy sleep pattern). A total of 208 patients (50.0%) had a healthy sleep pattern. Table 1A shows that patients with a healthier sleep pattern appeared to be more likely to have a lower body mass index (BMI); have a smaller size of the left atrium (LA); be more likely to have lower blood pressure, glucose and left atrial diameter; be less likely to have chronic diseases such as hypertension, diabetes, coronary artery disease and chronic heart failure.

**Table 1A:** Baseline characteristics of 416 patients according to quantities of sleep pattern score

	Healthy sleep score		
	0–1	2–3	4–5
Age, year	63.50 ± 7.99	63.65 ± 9.55	63.15 ± 9.72
Women (%)	12 (60.0)	74 (39.4)	90 (43.3)
BMI, kg/m^2^	25.01 ± 3.47	25.31 ± 3.25	24.78 ± 2.96
SBP, mm Hg	130.75 ± 20.01	127.93 ± 18.37	127.99 ± 16.73
DBP, mm Hg	81.70 ± 12.14	77.82 ± 12.09	78.43 ± 12.34
Glucose, mmol/L	6.61 ± 2.72	5.91 ± 1.42	5.73 ± 1.28
LA, cm	4.23 ± 0.59	4.19 ± 0.52	4.07 ± 0.57
Aspirin (%)	4 (20.0)	34 (18.1)	34 (16.3)
β‐blocker (%)	8 (40.0)	79 (42.0)	75 (36.0)
ACEI/ARNI (%)	9 (45.0)	67 (35.6)	62 (29.8)
Spironolactone (%)	3 (15.0)	40 (21.3)	35 (16.8)
Statins (%)	6 (30.0)	74 (39.3)	67 (32.2)
Physical acticity, METs (min/week)	819.60 ± 685.38	2088.40 ± 967.37	1714.05 ± 1124.67
Current smoking (%)	4 (20.0)	43 (22.9)	39 (18.8)
Current alcohol intake (%)	1 (5.0)	21 (11.2)	22 (10.6)
Hypertension (%)	13 (65.0)	120 (63.8)	118 (56.7)
Type 2 diabetes (%)	4 (20.0)	40 (21.3)	15 (7.2)
Coronary artery disease (%)	7 (35.0)	53 (28.2)	49 (23.6)
Heart failure (%)	12 (60.0)	77 (41.0)	76 (36.5)
Nonpersistent AF (%)	15 (75.0)	122 (64.9)	142 (68.3)
Substrate modification(%)	3 (16.7)	77 (45.3)	71 (39.4)
Follow‐up (months)	25.40 ± 16.62	28.64 ± 17.85	32.42 ± 18.18
LR (%)	10 (50.0)	80 (42.6)	40 (19.2)
ER (%)	4 (20.0)	26 (13.8)	17 (8.2)
Total	20	188	208

*Note*: Values are mean ± *SD* or *n* (%), unless otherwise indicated.

Abbreviations: ACEI, angiotensin converting enzyme inhibitor; AF, atrial fibrillation; ARNI, angiotensin receptor neprilysin inhibitor; BMI, body mass index; DBP, diastolic blood pressure; ER, early recurrence; LA, diameter of left atrium; LR, late recurrence; MET, metabolic equivalent; SBP, systolic blood pressure.

### 3.2 Characteristics of patients with recurrence

Characteristics of patients with AF recurrence are concluded in Table 1B. Among 416 participants, we documented 130 patients (31.3%) with an incidence of AF recurrence. The observed early recurrence of AF was 36 (26.9%) and 12 (4.2%) in patients without and with clinical recurrence, respectively (*p* < .01). According to Table 1B, women and patients with higher BMI are more likely to have a recurrence of atrial arrhythmia, though the difference between the two groups is not significant. Patients with recurrence have a lower healthy sleep pattern score of 2.93 ± 1.13, which is lower than that of patients without recurrence (3.67 ± 1.11, *p* < .01). As is shown in Table S1, patients with recurrence are more likely to need substrate modification such as ablation of the tricuspid‐valve isthmus, mitral‐valve isthmus and “BOX” ablation; are more likely to convert to sinus rhythm spontaneously during isolation of pulmonary veins; less likely to need electrical cardioversion during the ablation procedure.

**Table 1B:** Baseline characteristics of patients according to recurrence

	No‐recurrence (286)	Recurrence (130)	*p*‐value
Age <65 (%)	134 (46.9)	55 (42.3)	*p* = .388
Women (%)	130 (45.5)	46 (35.4)	*p* = .540
BMI, kg/m^2^	25.08 ± 3.16	24.87 ± 3.08	*p* = .515
SBP, mm Hg	128.60 ± 18.40	126.98 ± 15.77	*p* = .387
DBP, mm Hg	77.86 ± 12.13	79.29 ± 12.55	*p* = .271
Glucose, mmol/L	5.88 ± 1.53	5.80 ± 1.24	*p* = .577
LA, mm	4.09 ± 0.55	4.23 ± 0.55	*p* = .013
Healthy sleep pattern score	3.67 ± 1.11	2.93 ± 1.13	*p* < .01
Aspirin (%)	57 (19.9)	15 (11.5)	*p* = .036
β‐blocker (%)	113 (39.5)	49 (37.7)	*p* = .724
ACEI/ARNI (%)	99 (34.6)	39 (30.0)	*p* = .354
Spironolactone (%)	47 (16.4)	31 (23.8)	*p* = .073
Statins (%)	96 (33.6)	51 (39.2)	*p* = .263
Physical acticity, METs (min/week)	944.86 ± 1467.10	1245.85 ± 2512.91	*p* = .126
Current smoking (%)	54 (18.9)	32 (24.6)	*p* = .181
Current alcohol intake (%)	32 (11.2)	12 (9.2)	*p* = .547
Hypertension (%)	175 (61.2)	76 (58.5)	*p* = .598
Type 2 diabetes (%)	41 (14.3)	18 (13.8)	*p* = .894
Coronary artery disease (%)	80 (28.0)	29 (22.3)	*p* = .223
Heart failure (%)	39 (13.6)	26 (20.0)	*p* = .098
Nonpersistent AF (%)	203 (71.0)	76 (58.5)	*p* = .012
Substrate modification (%)	100 (35.0)	65 (50.0)	*p* = .04
ER	12 (4.2)	36 (26.9)	*p* < .01

*Note*: Values are mean ± SD or n (%), unless otherwise indicated.

Abbreviations: ACEI, angiotensin converting enzyme inhibitor; AF, atrial fibrillation; ARNI, angiotensin receptor neprilysin inhibitor; BMI, body mass index; DBP, diastolic blood pressure; ER, early recurrence; LA, diameter of left atrium; LR, late recurrence; MET, metabolic equivalent; SBP, systolic blood pressure.

### 3.3 Predictors of AF recurrence

**Figure 1:**
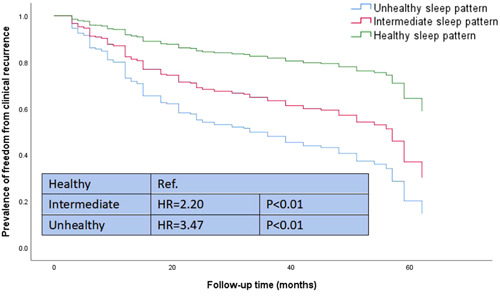
Cox regression analysis for prediction of AF recurrence. Cox regression analysis comparing the intermediate (red curve), unhealthy sleep pattern (green curve) to healthy sleep pattern (green curve), respectively. AF, atrial fibrillation

The association between sleep patterns and the risk of AF recurrence is shown in Figure 1. Sleep pattern was significantly associated with the risk of recurrent AF (*p* < .001). After being adjusted by different factors, an unhealthy sleep pattern was still significantly associated with AF recurrence [HR = 3.47, 95% CI (1.73–6.98), *p* < .001] when compared to a healthy sleep pattern. Intermediate sleep pattern was also observed to be associated with AF recurrence [HR = 2.20, 95% CI (1.14–2.30), *p* < .001] (Table 2A).

**Table 2A:** Cox regression analysis for prediction of AF recurrence

	Univariable analyses		Mutivariable analyses	
Variables	HR	*p*‐value	HR	*p*‐value
Male	0.72	0.142		
Age <65	0.9	0.538		
Hypertension	0.9	0.540		
Type 2 diabetes	0.97	0.902		
CAD	0.81	0.307		
Chronic HF	1.34	0.181		
LAD ≤40 mm	1.63	0.009		
Exercise	0.801	0.371		
Substrate modification	1.45	0.037		
AF type	1.618	0.007	1.62	.007
Β‐blocker	1.02	0.927		
ACEI/ARNI	0.88	0.494		
Spironolactone	1.39	0.112		
Sleep pattern				
Healthy	Ref.			
Intermediate	2.26	<0.01	2.20	<.01
Unhealthy	3.64	<0.01	3.47	<.01
ER	4.395	<0.01	4.41	<.01

Abbreviations: ACEI, angiotensin converting enzyme inhibitor; AF, atrial fibrillation; ARNI, angiotensin receptor neprilysin inhibitor; BMI, body mass index; CAD, coronary heart disease; DBP, diastolic blood pressure; ER, early recurrence; HF, heart failure; HR, hazard ratio; LA, diameter of left atrium; LR, late recurrence; MET, metabolic equivalent; SBP, systolic blood pressure.

The relationship between the index score of the healthy sleep pattern and risk of each outcome was generally similar across subgroups by age (<65 or ≥65 years), AF type, gender, BMI categories (without obesity, and obesity), smoking status, drinking status (Figure S2).

### 3.4 Different sleep behaviors and AF recurrence

**Figure 2:**
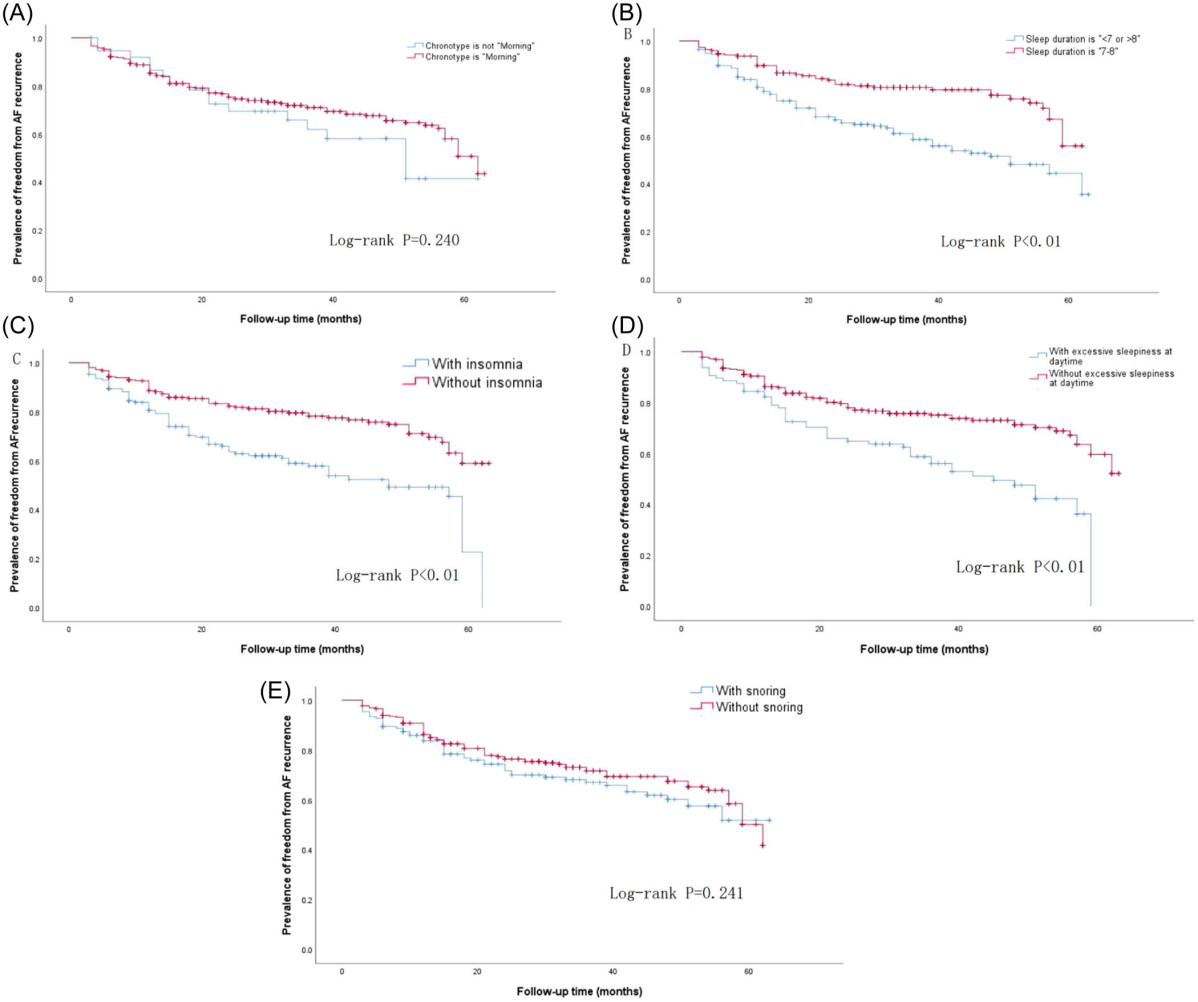
Kaplan–Meier curve comparing the clinical outcomes of different sleep behaviors. Kaplan–Meier estimation of the time to AF recurrence after ablation in patients with different sleep behaviors. (A) Chronotype of sleep; (B) Sleep duration; (C) Insomnia; (D) Excessive sleepiness at daytime; (E) Snoring

Figure 2 showed the results of the Kaplan–Meier estimation of the time to AF recurrence postprocedure in patients with different sleep behaviors. After adjusted by different factors, parameters such as “adequate sleep duration” [HR = 0.53, 95% CI (0.36–0.79)], “No insomnia” [HR = 0.47, 95% CI (0.32–0.68)] and “No excessive daytime sleepiness” [HR = 0.61, 95% CI (0.41–0.89)] were significantly associated with lower risk of AF recurrence, while “Morning chronotype” and “No snoring” was not associated with recurrent AF (Table 2B).

**Table 2B:** Association between five sleep behaviors and recurrence of AF

Sleep behaviors	No‐recurrence (286)	Recurrence (130)	Univariable analyses		Multivariable analyses	
			HR	95% Confidence interval	HR	95% Confidence interval
“Morning” chronotype	264 (92.3)	114 (87.7)	0.94	0.55–1.60		
Sleep duration is “7–8 h/d”	174 (60.8)	48 (36.9)	0.58	0.40–0.84	0.53	0.36–0.79
Without insomnia	187 (65.4)	59 (45.4)	0.52	0.36–0.74	0.47	0.32–0.68
Without snoring	185 (64.7)	79 (60.8)	0.81	0.57–1.15		
Without daytime sleepiness	239 (83.6)	81 (62.3)	0.64	0.44–0.93	0.61	0.41–0.89

*Note*: Association between different sleep behaviors and recurrent AF was performed in both univariable and multivariable Cox regression analyses. Healthy sleep pattern score was not included in models and other included covariates were the same as those in Table 2A.

Abbreviations: ACEI, angiotensin converting enzyme inhibitor; AF, atrial fibrillation; ARNI, angiotensin receptor neprilysin inhibitor; BMI, body mass index; CAD, coronary heart disease; DBP, diastolic blood pressure; ER, early recurrence; HF, heart failure; HR, hazard ratio; LA, diameter of left atrium; LR, late recurrence; MET, metabolic equivalent; SBP, systolic blood pressure.

### 3.5 Improved sleep preprocedure and AF recurrence

A total of 72 patients had a history of longer use of hypnotics and a total of 55 (76.4%) successfully improved their sleep quality before the procedure. Demographic characteristics were concluded in Table S2. The average sleep pattern score of the “Effectiveness Group” was significantly higher (3.76 ± 1.16 vs. 2.18 ± 1.13, *p* < .01) preablation. A total of 34 (61.8%) patients in the “Effectiveness Group” had a healthy sleep pattern preprocedure, while 16 patients (94.1%) in the “Failure Group” had an intermediate or unhealthy sleep patterns before ablation (Table S3).

Kaplan–Meier curve presented patients in “Effectiveness Group” were less likely to come down with recurrent AF when compared to those with unhealthy sleep pattern (including 6 patients in the “Failure Group”) (log‐rank *p* = .008) (Figure S3). No significant difference between the “Effectiveness Group” and “Healthy Sleep Pattern Group” was observed.

## 4 DISCUSSION

This retrospective study explored the association between sleep patterns and AF recurrence after CA. The results indicate that: (1) a healthy sleep pattern was associated with fewer episodes of recurrent AF; (2) three sleep behaviors (adequate sleep duration, no insomnia, and no excessive daytime sleepiness), rather than “Morning chronotype” and “No snoring,” were associated with lower risk of recurrence; (3) improved sleep before ablation was associated with lower risk of AF recurrence.

CA is an effective approach to achieving sinus rhythm for AF patients. However, many factors are associated with the failure of ablation.^16^ Apart from previously identified parameters associated with fibrosis of LA, some risk factors resulting in the activation of the automatic nervous system draw increasing attention. Sleep disorder is receiving more focus as it is closely related to daily life. However, sleep itself is very complicated and different sleep parameters are intrinsically correlated. Sleep patterns including five different behaviors can be used to evaluate sleep quality easily and quickly. However, the sleep pattern index score is subjective, while the bands in this questionnaire are easier to recall and can lead to less recalling bias when compared to PSQI.

For the first time, our study explored the association of overall sleep patterns evaluated by “Sleep pattern index score” with the risk of AF recurrence after CA. This new metric system is not clinically used but has been validated to be associated with episodes of different cardiovascular diseases and arrhythmias.^14^, ^15^, ^17^ Due to a limited number of patients, we divided patients into three groups according to their self‐reported score (0–1, 2–3, 4–5) when we explored the influence of sleep pattern on AF recurrence, which is different from previous research.^14^ Kim et al.^9^ found that improved sleep quality resulting from ablation was associated with a lower risk of recurrent AF in patients with non‐persistent AF, and sleep instability may be a predictor of recurrence. However, this study focused much more on the impact of alternation of sleep quality resulting from ablation, rather than whether sleep quality was improved before the procedure. Our study emphasized the association of sleep pattern pre‐procedure with AF recurrence. Considering the result of the subgroup analysis, we did not perform further analysis according to AF type. To weaken the impact of ablation to sleep patterns, we excluded patients reporting an obvious sleep instability after the procedure, which could decrease the bias in our research to some extent. Also, the exclusion of patients reporting sleep instability after ablation makes the interpretation of data easier based on the assessment of sleep patterns only once before CA.

Of different sleep disorders, obstructive sleep apnea (OSA) featuring snoring and excessive daytime sleepiness has been most studied. A meta‐analysis showed that untreated OSA can increase the risk of AF recurrence after CA.^18^ However, one RCT which was performed to explore the influence of treatment of OSA on AF recurrence showed no difference in recurrence in the two groups.^19^ The potential reason may be a small sample size (*N* = 25). Also, snoring is only a surrogate of OSA and the true impact of snoring on recurrence may not be equal to that of OSA. In our study, we explored the association between the parameter of 5 pooled behaviors and the risk of AF recurrence, and we found snoring and chronotype did not influence recurrence postprocedure. Previous studies showed that snoring was not associated with the incidence of AF, which may support our study results.^14^, ^20^

Our study also explored the association between improvement in sleep preablation and the risk of AF recurrence. Considering that evaluating sleep pattern based on score twice a time could cause more recalling bias, we judged sleep pattern was improved based on recorded case history or self‐reported results. Results showed that patients who improved their sleep pattern successfully pre‐procedure had a lower risk of recurrence when compared to those with unhealthy sleep patterns. We hence set a hypothesis that improved sleep prior can reduce the risk of recurrent AF and sleep pattern score might be a useful tool to evaluate and guide sleep therapy before ablation. One potential mechanism can support this hypothesis: better sleep patterns can reduce the activation of the autonomic nerve, which will influence both trigger of AF and atrial substrate preprocedure. Remarkably, one patient with a healthy sleep pattern had a history of longer using hypnotics. We checked her medical history carefully and found she took hypnotics due to a complaint of short sleep duration (about 6 h/day). However, hypnotics did not improve this symptom well.

Though our study retrospectively explored the association of sleep and recurrence after ablation, we took different sleep behaviors as a whole, which has been proven to be critical for the research associated with sleep influence.^14^, ^15^ Some potential mechanisms could explain the association between sleep patterns and AF recurrence observed in our study. It has been shown that sleep deprivation may disturb the autonomic nervous balance of sympathetic nervous and vagal outflows, which has been associated with induction and sustained arrhythmias.^14^, ^21^ Also, sleep can affect a brand range of metabolic changes such as lipid, glucose levels, blood pressure and oxidative stress, which would also increase recurrence risk.^22^, ^23^, ^24^ Additionally, various sleep behaviors may affect the development of cardiac arrhythmias via different and complementary pathways, so it is not surprising that their associations with recurrence exhibit an additive fashion when analyzed as a unit in the sleep pattern, as observed in our study.

## 5 LIMITATIONS

This study has several limitations. Since this retrospective study was conducted in a single center, selection bias and recalling bias are inevitable. AF recurrence may be underestimated due to incomplete capture. Also, an assessment of sleep patterns was only conducted once before ablation. We did not dynamically assess sleep patterns postprocedure, though patients reporting sleep instability postprocedure were excluded. For those who have a history of longer‐use of hypnotics, the intervention was monitored in the outpatient clinic, and we did not assess their initial sleep pattern. Second, there are many other more objective ways of monitoring sleep quality. However, we did not use them in this research setting because most patients were followed up in the form of phone interviews. As we mentioned before, though evaluation of sleep patterns is subjective, bands of the questionnaire are easy to be recalled and can cause less recalling bias. Third, the number of participants was limited, which could weaken the power of evidence. Finally, since the effect of hypnotics was evaluated based on medical history documented in anamnesis or self‐reporting results rather than being monitored in the same way, evidence from this study was not powerful enough to prove sleep pattern was a specific causation. However, we set a hypothesis that the possibility that sleep pattern can predict AF recurrence and our findings could be the basis for further research to explore the causality of sleep patterns. A well‐designed prospective research including larger samples is demanded to confirm these findings.

## 6 CONCLUSIONS

This retrospective study indicates that a healthy sleep pattern is associated with a lower risk of AF recurrence. Also, improvement in sleep before ablation is associated with a lower risk of recurrence. Our results support the hypothesis that better sleep patterns could reduce the recurrence of AF, which still requires more well‐designed studies to validate.

## AUTHOR CONTRIBUTIONS

**Jiehui Cang and Long Chen**: designed the study. **Jiehui Cang and Didi Zhu**: collected data. **Jiehui Cang and Naiyang Shi**: performed data analysis. **Jiehui Cang**: drafted article. **Yaowu Liu**: revised manuscript. All authors agree the submission of this manuscript.

## CONFLICTS OF INTEREST

The authors declare no conflicts of interest.

## Supporting information

Supporting information.

Supporting information.

## Data Availability

Immediately following publication, the deidentified participant all‐calculated data that support the findings of this study will be shared upon request. In addition, the data can be applicable to any type of analyses, and they will be shared using methods such as Excel or CSV files via Email. Please contact the corresponding author directly to request data sharing.
