# Supplementary material for: Self‐reported sleep pattern and recurrence of atrial fibrillation after catheter ablation
Source: Clin Cardiol. 2023 Jan 17;46(3):336–44. doi: 10.1002/clc.23975 (PMC10018108; doi:10.1002/clc.23975)
Supplement: Supplementary file 1 — Supporting information. [file CLC-46-336-s001.pdf]

Figure S1. Flow chart of screening process.

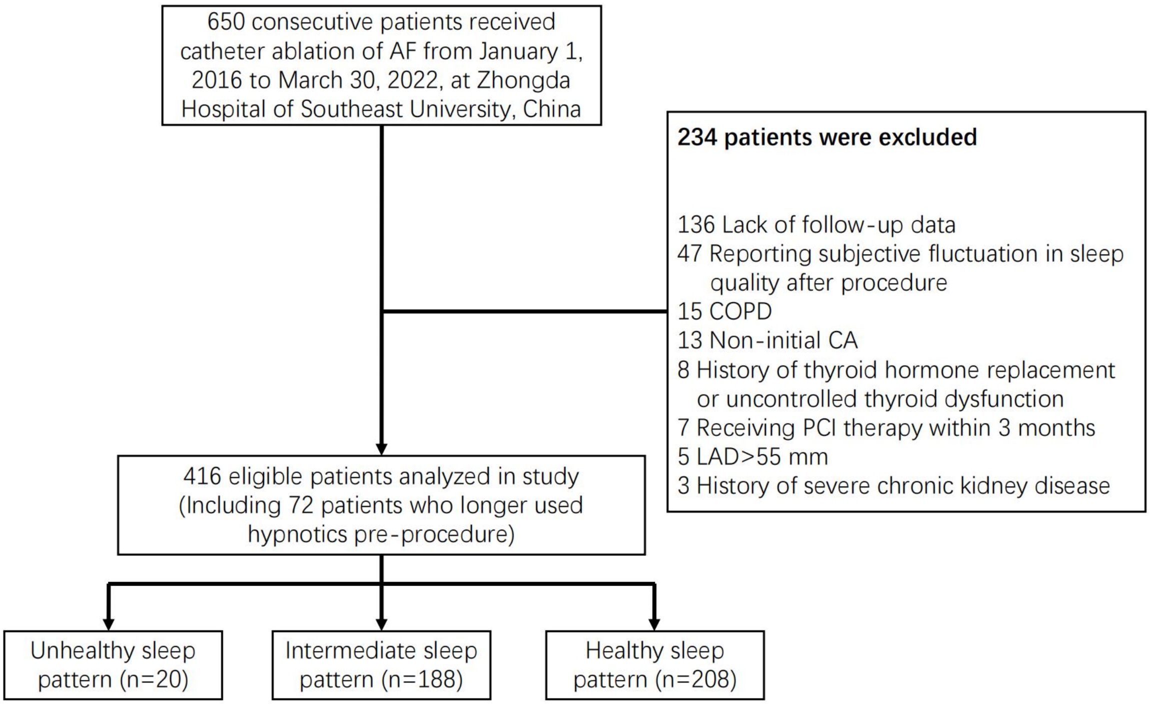

CA=catheter ablation; PCI=percutaneous coronary intervention.

Figure S2. Multivariable-Adjusted HRs for AF recurrence

Subgroups	Hazard Ratio (95% CI)	P-int
Gender		0.191
Woman	2.06 (1.43-2.97)	
Man	2.25 (1.35-3.50)	
Age		0.957
≤65	2.17 (1.49-3.15)	
>65	2.05 (1.35-3.10)	
CAD		0.38
No	2.09 (1.52-2.87)	
Yes	2.30 (1.29-4.11)	
Hypertension		0.321
No	2.77 (1.79-4.28)	
Yes	1.75 (1.21-2.51)	
Chronic heart failure		0.074
No	2.03 (1.48-2.78)	
Yes	2.18 (1.20-3.96)	
Type of AF		0.575
NPAF	2.25 (1.58-3.20)	
PAF	1.83 (1.16-2.91)	
Current smoking		0.598
No	2.18 (1.59-3.00)	
Yes	1.76 (1.00-3.10)	
Current drinking		0.695
No	2.19 (1.65-2.91)	
Yes	1.03 (0.36-2.97)	
BMI		0.659
Obesity	2.87 (1.79-4.58)	
Without Obesity	1.80 (1.27-2.49)	
Substrate modification		0.109
No	2.62 (1.74-3.96)	
Yes	1.70 (1.17-2.49)	
β-blocker		0.412
No	2.43 (1.69-3.48)	
Yes	1.72 (1.10-2.69)	
ACEI/ARNI		0.216
No	2.14 (1.52-3.01)	
Yes	2.08 (1.28-3.82)	
Spironolactone		0.526
No	2.13 (1.56-2.91)	
Yes	1.97 (1.06-3.64)	
ER		0.093
No	2.17 (1.58-2.99)	
Yes	1.34 (0.75-2.39)	
LAD		0.322
≤40mm	2.52 (1.54-4.14)	
>40mm	1.84 (1.32-2.57)	
Exercise		0.02
<700 METs	2.16 (1.40-3.35)	
700-1400 METs	2.30 (1.49-3.54)	
>1400 METs	1.88 (0.92-3.85)	

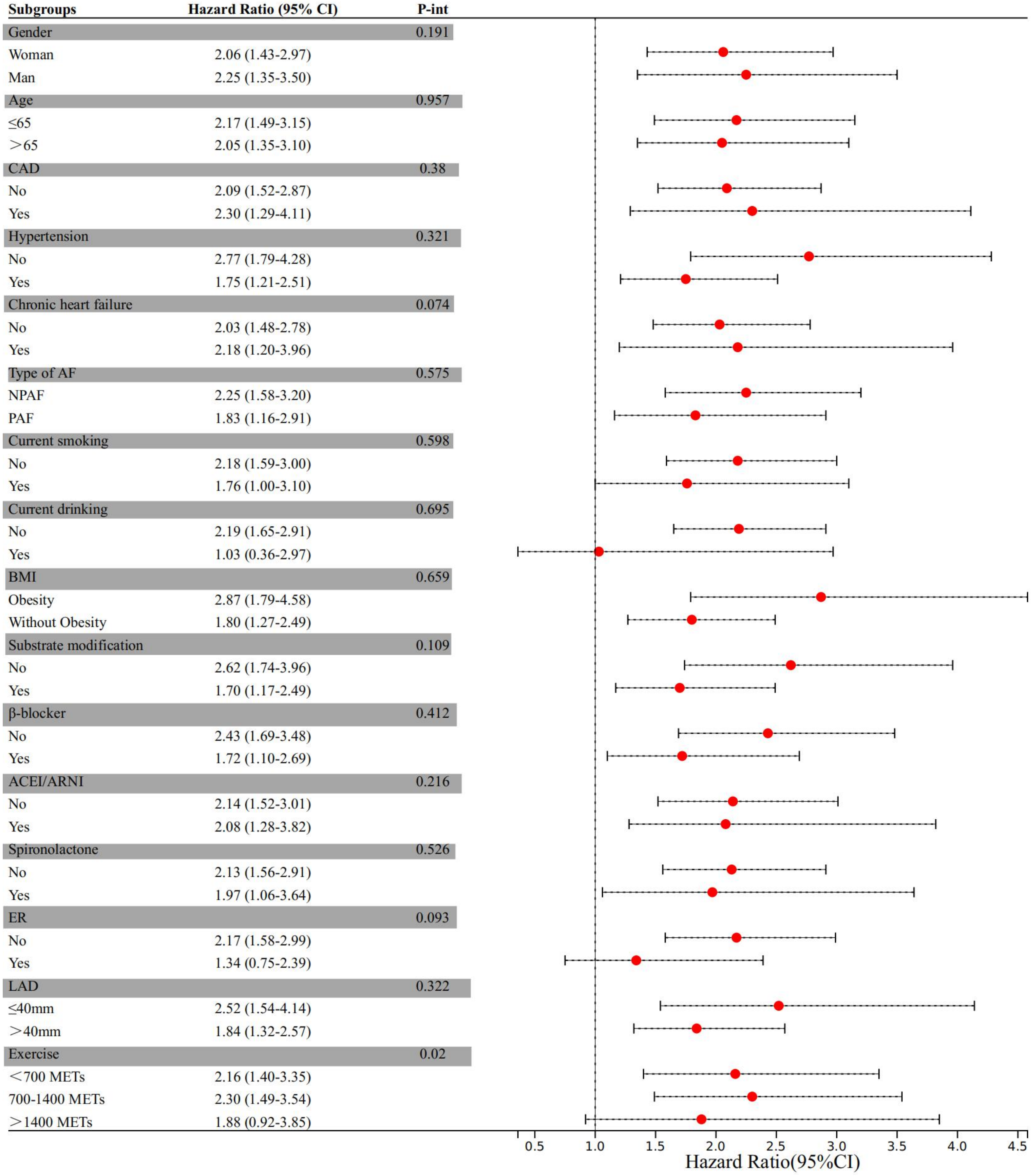

Stratified analysis was performed according to subgroups of each covariate. The interaction term of healthy sleep score with each potential modifier was included in the model. Abbreviations are described in **Table 1**.

Figure S3. Kaplan- Meier curve comparing difference between patients with hypnotics and patients without hypnotics.

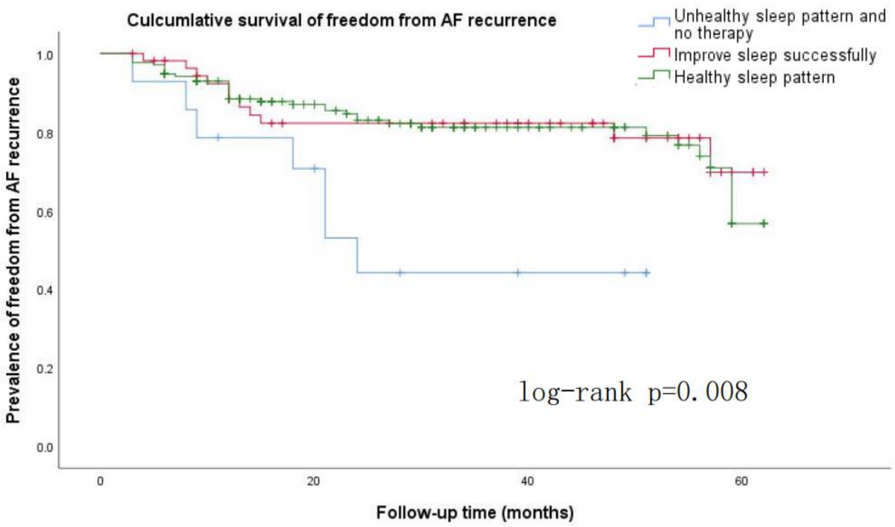

Kaplan-Meier estimation of the time to AF recurrence after ablation in: (1)patients who successfully improved sleep with sleep pattern score increasing more than one score (red curve); (2) patients with unhealthy sleep pattern and taking no sleeping pill pre-procedure (blue curve); (3) patients with healthy sleep pattern (green curve).
