# Supplementary material for: Self‐reported sleep pattern and recurrence of atrial fibrillation after catheter ablation
Source: Clin Cardiol. 2023 Jan 17;46(3):336–44. doi: 10.1002/clc.23975 (PMC10018108; doi:10.1002/clc.23975)
Supplement: Supplementary file 2 — Supporting information. [file CLC-46-336-s002.pdf]

Table S1. Procedural characteristics according to recurrence

		No-recurrence (286)	Recurrence (130)
Success of PVI (%)		286（100.0）	130（100.0）
Non-PV trigger	SVC	9（3.1）	5（3.8）
	FO	3（1.0）	0（0）
TVI (%)		55（19.2）	40（30.8）
MVI (%)		34（11.9）	28（21.5）
CAFE (%)		2（0.7）	2（1.5）
Superior/roof lesion (%)		55（19.2）	40（30.8）
Bottom lesion (%)		20（7.0）	25（19.2）
Anterior lesion (%)		7（2.4）	3（2.3）
Modification of LVA (%)		2（0.7）	2（1.5）
Electrical cardioversion (%)		76（26.8）	48（36.9）
Conversion to SR during PVI (%)		17（5.9）	6（4.6）
Total (%)		100（35.0）	65（50.0）

Values are mean±SD or n (%), unless otherwise indicated.

PVI= isolation of pulmonary veins; PV= pulmonary vein; SVC= superior vena cava; FO= fossa ovalis; TVI= isthmus of tricuspid valve; MVI= isthmus of mitral valve; CAFE= complex fractionated atrial electrograms; LVA= low-voltage area; SR= sinus rhythm.

Table S2. Characteristics of patients taking hypnotics.

	Failure Group	Effectiveness Group
Age, y	66.29±10.78	63.16±9.68
Women（%）	8 (47.1)	22 (40.0)
BMI, kg/m^2	23.28±2.42	25.00±3.13
SBP, mmHg	139.0±28.07	129.55±16.43
DBP, mmHg	82.24±14.53	77.58±12.08
Glucose, mmol/L	6.53±1.94	5.97±1.76
LA, cm	4.08±0.50	4.12±0.58
Aspirin (%)	5 (29.4)	12 (23.6)
β-blocker (%)	10 (58.8)	24 (43.6)
ACEI/ARNI (%)	6 (35.3)	15 (27.3)
Spironolactone (%)	2 (11.8)	13 (23.6)
Statins (%)	7 (41.2)	27 (49.1)
Physical acticity, METs (min/week)	698.00±693.88	938.40±1084.55
Current smoking (%)	4 (23.5)	11 (20.0)
Current alcohol intake (%)	2 (11.8)	10 (18.2)
Hypertension (%)	11 (64.7)	29 (52.7)
Type 2 diabetes (%)	3 (17.6)	7 (12.7)
Coronary artery disease (%)	8 (47.1)	15 (27.3)
Heart failure (%)	2 (11.8)	14 (25.5)
Non-persistent AF (%)	2 (11.8)	13 (23.6)
Substrate modification(%)	8 (47.1)	28 (50.9)
Follow-up (months)	30.71±17.53	36.29±19.31
LR (%)	6 (35.3)	11 (20.0)
ER (%)	3 (17.6)	5 (9.1)
Total	17	55

Values are mean±SD or n (%), unless otherwise indicated.

BMI= body mass index; SBP= systolic blood pressure; DBP= diastolic blood pressure; LA= diameter of left atrium; MET= metabolic equivalent; ACEI= Angiotensin Converting Enzyme inhibitor; ARNI= Angiotensin Receptor Neprilysin inhibitor; AF= atrial fibrillation;LR=late recurrence; ER=early recurrence.

Table S3. Distribution of scores and 5 sleep behaviors according with effect of hypnotics.

	Failure Group	Effectiveness Group	*P* value
Score of sleep pattern after therapy of hypnotics			
5	0 (0)	12 (21.8)	
4	1 (5.9)	22 (40.0)	
3	8 (47.1)	17 (30.9)	
2	2 (11.8)	4 (7.3)	
1	5 (29.4)	0 (0)	
0	1 (5.9)	0 (0)	
Average score (Mean±SD)	2.18±1.13	3.76±1.16	<0.01
Different sleep behaviors			
"Morning" chronotype	12 (70.6)	50 (90.9)	0.049
Sleep duration is "7-8h/d"	6 (35.3)	35 (63.6)	0.039
No insomnia	4 (23.5)	35 (63.6)	0.005
No snoring	7 (41.2)	37 (67.3)	0.086
No excessive daytime sleepiness	8 (47.1)	50 (90.9)	<0.01
